# Reliability of Quadruplicated Serological Parameters in the Korean Genome and Epidemiology Study

**DOI:** 10.4178/epih/e2011004

**Published:** 2011-05-19

**Authors:** Jae Jeong Yang, Ji Hyun Yang, Jimin Kim, Lisa Y. Cho, Boyoung Park, Seung Hyun Ma, Sang Hoon Song, Won-Ki Min, Sung Soo Kim, Man Suck Park, Sue K. Park

**Affiliations:** 1 Department of Preventive Medicine, Seoul National University College of Medicine, Seoul, Korea.; 2 Cancer Research Institute, Seoul National University, Seoul, Korea.; 3 Center for Genome Science, Korea National Institute of Health, Osong, Korea.; 4 Department of Laboratory Medicine, Seoul National University College of Medicine, Seoul, Korea.; 5 Department of Laboratory Medicine, Asan Medical Center, University of Ulsan College of Medicine, Seoul, Korea.; 6 Department of Biomedical Science, Seoul National University Graduate School, Seoul, Korea.

**Keywords:** Reliability, Statistical compensation model, Serological marker, The KoGES

## Abstract

**OBJECTIVES:**

The aim of this study was to evaluate whether clinical test values from different laboratories in the Korean Genome and Epidemiology Study (KoGES) can be integrated through a statistical adjustment algorithm with appropriate intra- and inter-laboratory reliability.

**METHODS:**

External quality control data were obtained from the Korean Society for Laboratory Medicine and quadruplicated standardized serological samples (N=3,200) were manufactured in order to check the intra- and inter-laboratory reliability for aspartic acid transaminase (AST), alanine transaminase (ALT), gamma-glutamyl transpeptidase (γ-GTP), blood urea nitrogen (BUN), creatinine, uric acid (UA), fasting blood sugar (FBS), cholesterol, and triglyceride (TG). As an index of inter- and intra-rater reliability, Pearson's correlation coefficient, intraclass correlation coefficients and kappa statistics were estimated. In addition, to detect the potential for data integration, we constructed statistical compensation models using linear regression analysis with residual analysis, and presented the R-square values.

**RESULTS:**

All correlation coefficient values indicated good intra- and inter-laboratory reliability, which ranged from 0.842 to 1.000. Kappa coefficients were greater than 0.75 (0.75-1.00). All of the regression models based on the trial results had strong R-square values and zero sums of residuals. These results were consistent in the regression models using external quality control data.

**CONCLUSION:**

The two laboratories in the KoGES have good intra- and inter-laboratory reliability for ten chemical test values, and data can be integrated through algorithmic statistical adjustment using regression equations.

## INTRODUCTION

The Korean Genome and Epidemiology Study (KoGES), a large population-based genomic cohort study supported by the Korea Centers for Disease Control and Prevention (KCDC), investigates risk factors for major diseases among Koreans, in particular focusing on gene-environment and gene-gene interactions [1,2]. According to the standardized KoGES protocol 12 biochemical analyses (fasting blood sugar [FBS]; gamma-glutamyl transpeptidase [γ-GTP]; aspartic acid transaminase [AST]; alanine transaminase [ALT]; total cholesterol; triglyceride [TG]; high density lipoprotein [HDL]-cholesterol; low density lipoprotein [LDL]-cholesterol; albumin; blood urea nitrogen [BUN]; uric acid [UA]; and creatinine), complete blood cell counts including eight hematologic indices (white blood cells [WBC]; red blood cells [RBC]; hemoglobin [Hb]; hematocit [HCT]; mean corpuscular volume [MCV]; mean corpuscular hemoglobin [MCH]; mean corpuscular hemoglobin concentration [MCHC]; and platelets), and analysis of high sensitivity c-reactive protein (HS-CRP) was conducted at two clinical laboratories, and authenticated via external quality assessment (QA) by the Clinical and Laboratory Standards Institute (CLSI) and the Korean Society for Laboratory Medicine (KSLM).

Clinical results from multiple laboratories may differ systematically [3-5]. Despite standardization of the blood test in the KoGES, potential discrepancies that may lead to significantly biased results in the pooled analyses remain. Moreover, the fundamental differences between the laboratories involving instruments (Hitachi Co., Tokyo, Japan vs. Bayer HealthCare Ltd., Tarrytown, NY, USA), methods (enzymatic vs. colorimetry for γ-GTP) and reference values (adapted to each instrument) may result in heterogeneity.

To minimize the potential analytic drift, appropriate standardization such as calibration or statistical adjustment is warranted [6]. Thus, we investigated whether 1) the KoGES laboratories measure consistent test values regardless of time and environmental conditions, 2) the laboratories are able to produce the same test values for future data pooling, and 3) future pooled analysis for serological parameters is possible using a statistical compensation model even though absolute test values differ between laboratories. In the present study, the intra- and inter-laboratory reliability of the two clinical laboratories were assessed using quadruplicated standardized serological samples and external quality control (QC) data, and the possibility that serological parameters produced by different laboratories can be integrated through statistical compensation models was evaluated.

## METHODS

To check the overall reliability of each laboratory, three years of QC data (2005, 2006, and 2007) for ten blood chemical tests (albumin, ALT, AST, γ-GTP, BUN, creatinine, UA, FBS, cholesterol, and TG) were obtained from the KSLM. To evaluate intra- and inter-laboratory reliability, quadruplicated standardized serological samples were prepared according to the Clinical and Laboratory Standards Institute (CLSI) guidelines [7]. Basic principles including 'at least 40 patient samples', 'at least 5 operating days', and 'analysis of each sample in duplicate within the same run' were adopted. The principle of 'at least 50% of samples outside the reference range' was revised based on the true range of each test result in the KoGES and the general distribution in the Korean population. Finally, 40 samples were divided into five groups with two outlier groups A and E (Table 1).

The standardized serological samples were manufactured at the Department of Laboratory Medicine at the Seoul National University College of Medicine. Eight anonymous patient samples were prepared for five consecutive operating days. During the repeated trial, a total of 3,200 test serological samples were manufactured (eight patients×five operating days×duplication×repeated trial×ten test items×two laboratories). In detail, sample preparation was carried out as follows: 1) a nurse at the Department of Laboratory Medicine selected all serum samples with a volume of more than 3 mL before the day of the test, 2) a well-trained clinical pathologist selected eight serum samples on the morning of the test, 3) four 250 µL aliquots of each serum (quadruplicated samples) were divided into plain tubes, and 4) a set of duplicated samples was delivered to each center. To minimize other sources of variation, sample delivery, storage conditions, and test time were consistent with a standardized protocol. On the day of the test, prepared samples were delivered to each laboratory between noon and 1 pm, and tests were conducted simultaneously at 3 pm. Serum selection and tests were not conducted on a Saturday, Sunday, or Monday. The first trial was conducted on November 29-30 and December 4-7, 2007. On the 6th day of the first trial, additional tests for FBS, BUN, UA, and albumin with unacceptable values outside the reference range (from Groups A or E) were conducted. The second trial was conducted over five days on December 11, 12, 13, 14, and 20, 2007.

The mean of the difference and the coefficient of variation (CV) between the duplicated test results at each laboratory were calculated to determine intra-rater reliability. Pearson's correlation coefficients and intra-class correlation (ICC) coefficients were estimated as indices of intra-rater reliability. To determine the inter-laboratory reliability, we estimated the mean and CV of the differences between the mean of 40 duplicated test values at Centers X and Y. The level of agreement between the two laboratories was assessed using Pearson's correlation coefficients, ICC coefficients and the Bland-Altman Plot [8]. Kappa statistics were used to measure the agreement in diagnosis (normal vs. abnormal). Coefficient values of 0.81-1.00 and >0.75 indicated 'almost perfect' and 'substantial and excellent', respectively [9,10].

Statistical compensation models were constructed using linear regression analysis to determine the potential for data integration. The regression equation was as follows: (Test values at Center Y)=α (intercept)+β (slope)×(Test values at Center X). The model fitting was checked by both regression residual analysis and R-square values. All statistical analyses were performed using SAS version 9.2 (SAS Inc., Cary, NC, USA) and MedCalc version 11.5 (MedCalc Software, Inc, Mariakerke, Belgium).

The present study was approved by the Institutional Review Boards of Seoul National University Hospital and the National Cancer Center of Korea (C-0707-072-214).

## RESULTS

**Figure 1:**
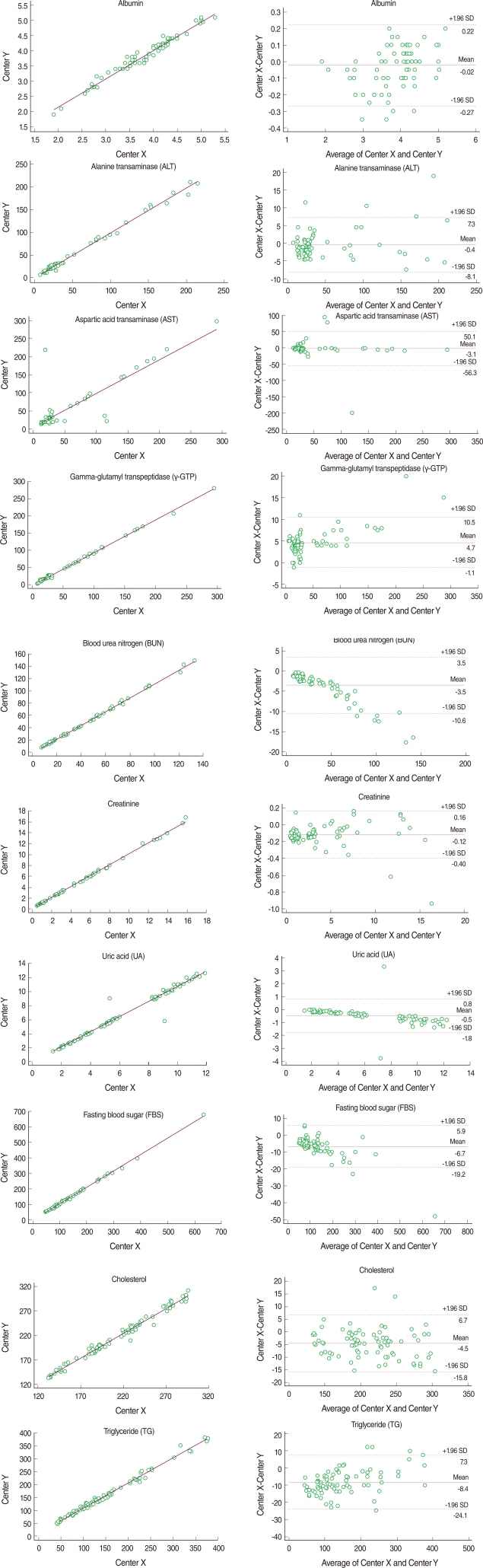
Scatter plots and Bland-Altman plots for each blood test item. SD, standard deviation.

For intra-rater reliability at Center X, the mean differences ranged from 0.027 to 7.387. With the exception of AST (0.842), all correlation coefficients were greater than 0.997 (p<0.001). The ICC coefficients indicated excellent reliability that was greater than 0.836. Similarly, the mean differences at Center Y ranged from 0.018 to 3.125, all Pearson's correlation coefficients ranged from 0.989 to 0.999, and ICC coefficients ranged from 0.990 to 1.000, indicating almost perfect intra-rater reliability. Inter-laboratory reliability between Center X and Y was highly comparable. The means of the absolute values of the differences between the mean of the 40 duplicated test values at Centers X and Y ranged from 0.095 to 9.737. All correlation coefficients were reliable. Though AST showed the lowest Pearson's correlation coefficient and an ICC coefficient equal to the intra-rater reliability, the values remained good at 0.872 and 0.871, respectively. The kappa statistics showed attenuated reliability but all values were over 0.75 with substantial reliability (Table 2). Bland-Altman plots showed that most absolute differences between Center X and Y were within the 95% limit of agreement (Figure 1).

Using the standardized serological samples, statistical compensation models using linear regression analysis were constructed to yield a mathematical relationship between the results of the two laboratories. With the exception of AST (R^2^=0.70), all regression equations presented strong correlation coefficients (R^2^>0.97). The regression equations for the three years, 2005, 2006, and 2007, were estimated using external QC data acquired from the KSLM. All correlation coefficient values were greater than 0.95 with the exception of TG, which used a different QC method in the two laboratories in 2005 and 2006. All the results of the standardized serological samples and external QC data were consistent in the regression models (Table 3).

In the residual analysis, the sums of residuals were all zero and for those residual plots that presented residuals between the actual y-values and the predicted values all residuals were randomly distributed around zero (data not shown).

## DISCUSSION

This study evaluated the possibility of integrating serological parameters from different laboratories participating in the KoGES via a statistical adjustment algorithm with good intra- and inter-laboratory reliability. Our results indicated that the two laboratories had excellent intra- (correlation coefficient>0.84, ICC>0.83) and inter-laboratory reliability (correlation coefficient>0.87, ICC>0.87, and kappa>0.75) for ten chemical test values. Moreover, linear regression analysis to compensate for the discrepancy in test values between the two centers gave excellent R-square values and zero sums of residuals.

Poorly controlled data can lead to significantly biased results [11]. Given the participation of two laboratories in the KoGES, the issue of quality control should be carefully addressed. Likewise, a strategy for data integration should be established based on the reliability within and between the laboratories. The present study indicated that regression equations with higher R-square values can compensate for the potential discrepancies caused by the use of different laboratories. Although the absolute values differed slightly, if intra- and inter-laboratory reliability can be assured, data integration may be successfully conducted using statistical compensation models. In terms of AST, γ-GTP and TG with relatively unstable results or insufficient external QC data, further replication studies focused on clinical features and test methodology are required.

The issue of multiple laboratories is relevant not only for the KoGES but also for other large cohorts. Ideally, a single laboratory that passes a strict QC system should conduct all clinical tests according to accurate and standardized methods. However, this is logistically difficult in reality. The most reasonable alternatives are 1) choosing reliable laboratories, 2) developing a standardized protocol for clinical tests, 3) conducting and monitoring regular QC, and 4) integrating the KoGES database after statistical adjustment. For statistical adjustment, the regression analysis method can be used to check intra- and inter- rater reliability.

Using the KoGES data, this study aimed to determine the possibility of integrating serological parameters produced from different laboratories via statistical compensation models. Our results indicate that the ten blood chemical tests analyzed at the two laboratories can be integrated through statistical adjustments using regression equations. The existing external QC data should be used to correct the discrepancies in the other biochemical tests and complete blood cell counts, or additional trials with the standardized serological samples should be conducted before data integration into the KoGES database.

## Figures and Tables

**Table 1:**
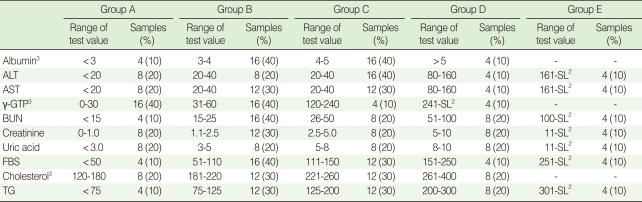
Total number of standardized serological samples (N=40) prepared according to the test value range^1^

ALT, alanine transaminase; AST, aspartic acid transaminase; γ-GTP, gamma-glutamyl transpeptidase; BUN, blood urea nitrogen; creatinine; UA, uric acid; FBS, fasting blood sugar; TG, triglyceride. ^1^Using 40 serum samples, the standardized serological samples were prepared from the number of specimens predetermined according to the range of test values. We prepared each sample twice, and thus 80 serum samples were acquired in this study; ^2^The maximum value that could be detected by each instrument; ^3^Albumin, γ-GTP, and cholesterol were divided into four categories based on both the KoGES data and normal ranges for the general Korean population.

**Table 2:**
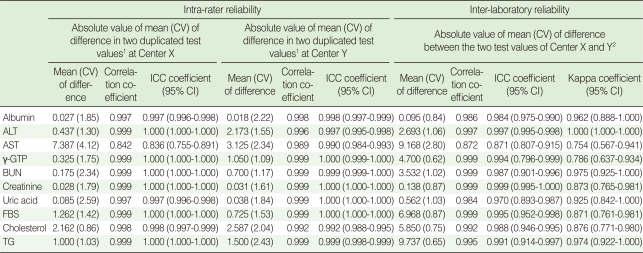
Intra-rater reliability at each center and inter-laboratory reliability between the two test values at Centers X and Y

ALT, alanine transaminase; AST, aspartic acid transaminase; γ-GTP, gamma-glutamyl transpeptidase; BUN, blood urea nitrogen; UA, uric acid; FBS, fasting blood sugar; TG, triglyceride; CV, coefficient of variation; ICC, intra-class correlation. ^1^Calculated as the absolute value of the difference in the mean of two duplicated test values at each center (n=80); ^2^Calculated as the absolute value of the difference in the mean of two duplicated test values at Center X (n=80) and the mean of two duplicated test values at Center Y (n=80).

**Table 3:**
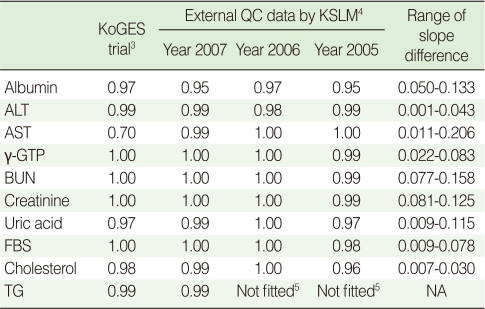
R-square values (sum of residuals)^1^ on the fitting of linear regression models^2^ using internal and external quality control data

ALT, alanine transaminase; AST, aspartic acid transaminase; γ-GTP, gamma-glutamyl transpeptidase; BUN, blood urea nitrogen; UA, uric acid; FBS, fasting blood sugar; TG, triglyceride; KoGES, Korean Genome and Epidemiology Study; KSLM, Korean Society for Laboratory Medicine; QC, quality control; NA, not applicable. ^1^Sums of residuals were all zero; ^2^Equation forms are represented by (value of Center Y)=α (intercept)+β (slope)×(value of Center X). ^3^The quadruplicated standardized samples from 40 samples; ^4^Three standardized samples were tested for external QC four times a year by the KSLM; ^5^Different QC methods applied in the two laboratories in 2005 and 2006.
